# Antifungal Activity of Gentamicin B1 against Systemic Plant Mycoses

**DOI:** 10.3390/molecules25102401

**Published:** 2020-05-21

**Authors:** Gaspar Banfalvi

**Affiliations:** Department of Molecular Biotechnology and Microbiology, University of Debrecen, 4010 Debrecen, Hungary; bgaspar@unideb.hu or gaspar.banfalvi@gmail.com; Tel.: +36-52-512-900 (ext. 62319); Fax: +36-52-512-925

**Keywords:** gentamicin, antifungal effect, pulmonary diseases, fusariosis, aspergillosis, synergism

## Abstract

Background: Gentamicin is a broad-spectrum aminoglycoside antibiotic produced by *Micromonospora purpurea* bacteria, effective against Gram-negative bacterial infections. Major fractions of the gentamicin complex (C1, C1a, C2, C2a) possess weak antifungal activity and one of the minor components (A, A1–A4, B, B1, X), gentamicin B1 was found to be a strong antifungal agent. Methods: This work uses in vitro and in vivo dilution methods to compare the antifusarial, antiaspergillic and anticryptococcal effects of gentamicin derivatives and structurally-related congeners. Results: The in vitro antifusarial activity of gentamicin B1 (minimum inhibitory concentration (MIC) 0.4 μg/mL) and structurally-related compounds (MIC 0.8–12.5 μg/mL) suggests that the purpuroseamine ring substituents are responsible for the specific antimycotic effect. The functional groups of the garoseamine and 2-deoxystreptamine rings of gentamicin derivatives are identical in gentamicin compounds and are unlikely to exert a significant antifungal effect. Among soil dermatophytes, *Microsporum gypseum* was more susceptible to gentamicin B1 (MIC 3.1 µg/mL) than *Trichophyton gypseum* (MIC 25 µg/mL). The in vitro antifungal effect of gentamicin B1 against plant pathogenic fungi was comparable to primary antifungal agents. Conclusion: Gentamicin is already in medical use. In vitro and preclinical in vivo synergisms of gentamicin B1 with amphotericin B suggest immediate clinical trials starting with subtoxic doses.

## 1. Introduction

Mycoses may cause not only mild allergic reactions but also life-threatening opportunistic pathogenic diseases such as invasive aspergillosis, fusariosis or candidiasis [1,2]. Noninvasive bronchopulmonary diseases may lead to invasive pulmonary aspergillosis not only in immunocompromised but even in immunocompetent individuals [3]. In the last few decades, the incidence of aspergillosis rose primarily due to infections with *Aspergillus fumigatus* and other pathogenic *Aspergillus* species [4].

Disseminated fusariosis is the second-most-frequent lethal fungal infection after aspergillosis, especially in neutropenic patients with hematologic malignancy [5]. Bronchopulmonary fusariosis occurs almost exclusively in severely immunocompromised persons, especially in acute leukemia patients and recipients of allotransplants [1]. These infections are difficult to treat because persons infected with endemic mycoses are immunosuppressed and resistant to antifungal agents [6]. The pulmonary aspects of cryptococcosis are often overlooked because the manifestation of cryptococcal infection is meningoencephalitis, although the initial pathogenetic event is pulmonary infection. *Cryptococcus gatti* and *Cryptococcus neoformans* are the etiologic agents causing major systemic pulmonary infections [7].

The attachment of alkyl groups to antibacterial aminoglycosides altered the antimicrobial properties of kanamycins and neomycins and inhibited the growth of fungi [8,9]. Amphiphilic kanamycins are antifungal, but not antibacterial, and inhibit the growth of fungi by interfering with the plasma membrane functions [8]. This is reflected by the attachment of hydrophobic residues such as linear alkyl chains on the aminoglycoside backbone. As a result of changes from antibacterial to antifungal character, novel mechanisms of action have been developed [9].

Gentamicin B1 turned out to be a low-toxicity antifungal agent against *Fusarium* species [5], and other plant pathogen fungi susceptible to gentamicin B1. These infections could be treated with gentamicin B1. The gentamicin complex is much less effective and exerted only a moderate antifungal effect against *Fusarium* species [5,10]. To reduce the antibiotic resistance and to optimize fermentation conditions, producers removed gentamicin from the solid waste and sewage water in gentamicin factories using biosorption and biodegradation by fungi [11,12] including molds like *Aspergillus niger* and *Aspergillus. terreus* [13].

The present study describes the strong antifungal effect of gentamicin B1 on plant and human pathogenic molds. Along with the measurement of in vitro minimum inhibitory concentration (MIC) values, synergy studies were performed by the combination of gentamycin B1 and amphotericin B. The severe nephrotoxicity of amphotericin B and the lack of antifungal antibiotics justify such new approaches.

## 2. Results

### 2.1. Antifusarial Effect of Gentamicins and Aminoglycoside Derivatives

**Figure 1:**
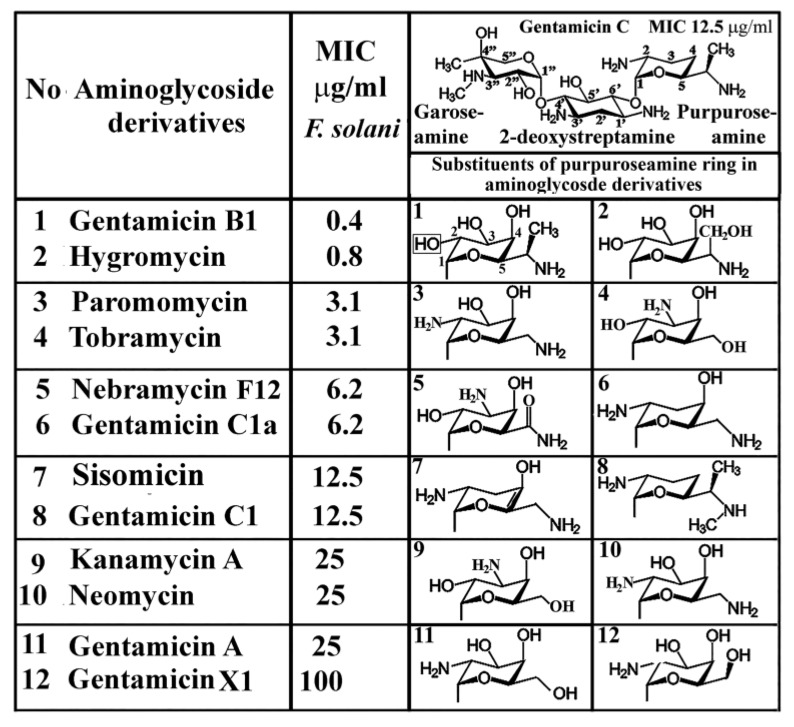
Structure–function relationship of aminoglycosides based on purpuroseamine ring substituents. In vitro antifungal activities are expressed as minimum inhibitory concentration (MIC) values in µg/mL against *Fusarium solani.* Technical details are given in the Methods. The figure was modified with permission [5].

In addition to gentamicin, the clinically available readthrough inducer, G418 has been recommended to counteract the effects of nonsense mutations in several genetic diseases and cancers [14,15]. This approach was discontinued when it turned out that the synthetic aminoglycoside G418, a closely related compound to gentamicin B1, was not an inducer of premature termination codon readthrough [16]. The substituents of gentamicin B1 and G418 (geneticin) differ only in the location of C2 of the purpuroseamine ring with a hydroxyl group in gentamicin B1 substituted by an amino group in G418. Other compounds also related to gentamicins and aminoglycoside derivatives turned out to be efficient inhibitors of the human pathogen *Fusarium solani* (Figure 1).

The purpuroseamine ring substituents of gentamicin C (upper panel of Figure 1) are present in aminoglycoside derivatives (hygromycin, paromomycin, neomycin) that consist not only of three but four rings. Irrespective of the number of rings, Figure 1 shows only the purpuroseamine ring substituents of aminoglycosides that are related to gentamicin. The presence of the 4th ring and its substituents do not impact the antifungal effect. The in vitro antifusarial spectrum of aminoglycosides (Figure 1) resolved several doubts related to the structure–function relationship of gentamicin. The fine-tuning of the antifusarium effect takes place through the substituents of the purpuroseamine ring of the gentamicin complex and not at the garoseamine and 2-deoxystreptamine units that are identical in each gentamicin structure.

### 2.2. Antifungal Agents against Plant Pathogenic Fungi

We tested whether the antifungal activity of gentamicin B1 on *Fusarium* species is an inherent property affecting other plant pathogen fungi such as *Cryptococcus, Fusarium, Microsporum, Trichophyton* or yeasts, e.g., *Candida* cultures. The antifungal activity of different agents aside from gentamicin B1, including clotrimazole, amphotericin B, nystatin and griseofulvin are summarized in Table 1. Some of the MIC values of gentamicin B1 for *Trychophyton* and *Candida albicans* are higher than the concentrations of amphotericin B and clotrimazole. Gentamicin B1 is more efficient against *Fusarium*, *Aspergillus*, *Microsporon and Cryptococcus* species than other established antifungal agents such as clotrimazole, amphotericin B, nystatin or griseofulvin.

The variability in antifungal effect depended not only on the chemical composition of components of gentamicin but also on the types of plant fungi tested. The soil dermatophyte *Microsporum gypseum* is often difficult to distinguish from *Trichophyton gypseum.* Based on its inhibition, *Microsporum gypseum* is more susceptible (MIC 3.1 µg/mL) to gentamicin B1 than *Trichophyton gypseum* (MIC 25 µg/mL) (Table 1). Based on this observation, gentamicin B1 could serve as a differential diagnostic tool to distinguish between these two species.

MIC values of antifungal agents were measured in different species of plant pathogenic fungi and *Candida albicans* yeast cells. Antifungal activities were determined by twofold serial dilutions of sterile Sabouraud broth fungal medium at pH 7.2 described in the Methods section.

MIC values of primary antifungal agents against different fungi vary significantly [17] and are demonstrated in Table 1. The MIC values of growth inhibition of *Fusarium* species by gentamicin B1 (0.2–3.1 µg/mL MIC values), clotrimazole (3.1–12.5 µg/mL) and amphotericin B (3.1–50 µg/mL) allow some ranking among antifungal compounds. In making this judgement, the functional groups in aminoglycosides, particularly those of the purpuroseamine ring of gentamicin B1 were taken into consideration (Table 1). The highest antifungal potential against different types of molds was attributable to clotrimazole, followed by gentamicin B1, amphotericin B, nystatin and griseofulvin. By comparing only the specific antifungal potential on systemic plant and soil pathogens including *Fusarium*, *Aspergillus*, *Cryptococcus* and *Microsporum* species, gentamicin B1 was the most efficient antifungal compound followed by clotrimazole and amphotericin B. Among plant pathogen molds, the invasiveness of *Fusarium solani* is underlined by its implication in about half of the systemic fusarioses causing, among others, disseminated diseases and fungemia in the blood of immunocompromised patients.

### 2.3. Synergy between Amphotericin B and Gentamicin B1 against C. Neoformans

*Cryptococcus* infections deserve attention as they have been increasing steadily since the onset of AIDS and by the expanded use of immunosuppressive drugs. *Aspergillus* species cause the most severe mold infections [18], but other plant pathogenic fungi, among them Cryptococcus, are of increasing concern. The anticryptococcal activities of gentamicin B1 and amphotericin B were determined separately and in combination to judge their potential beneficial interactions. Synergism is shown by a parabolic curve. Additive effects would be indicated by a linear and antagonism by a hyperbolic relationship.

**Figure 2:**
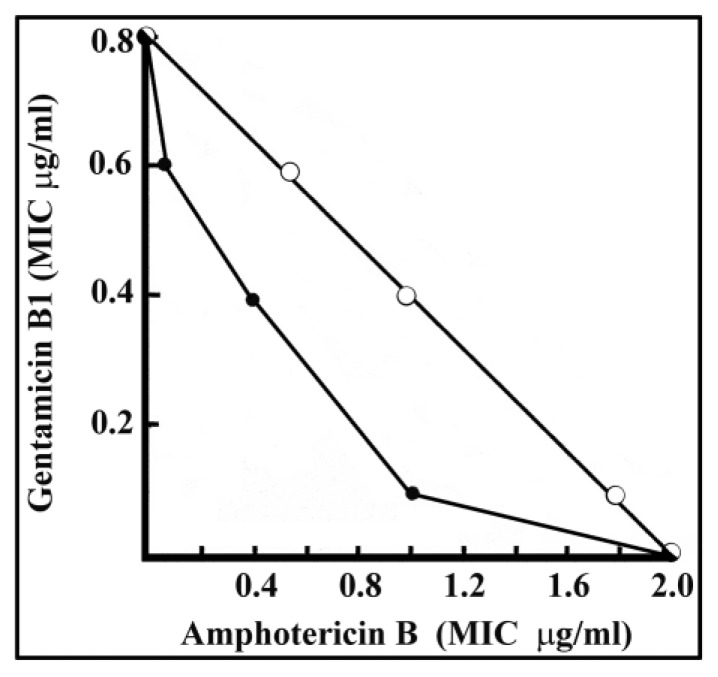
In vitro synergistic effect of amphotericin B combined with gentamicin B1 against *Cryptococcus neoformans.* The MIC values of gentamicin B1 alone (0.8 µg/mL) and amphotericin B alone (0.8 µg/mL) are shown as well as the combined action of the two compounds (-•-). The upper curve is plotted only to indicate theoretical MIC values without synergistic interaction (-o-). The parabolic curve shows the cooperative interaction of the two substances to produce a combined effect and lower MIC values.

The in vitro synergistic interaction between amphotericin B and gentamicin B1 against *C. neoformans* is seen in the parabolic curve (-•-) of Figure 2. The MIC values of amphotericin B alone (2 µg/mL) and gentamicin B1 by itself (0.8 µg/mL) are significantly higher than their synergistic combinations (Figure 2):

(a) 1 µg/mL amphotericin B and 0.1 µg/mL gentamicin B1,
(b) 0.4 µg/mL amphotericin B and 0.4 µg/mL gentamicin B1, or
(c) 0.1 µg/mL amphotericin B + 0.6 µg/mL gentamicin B1,

with the highest synergy obtained at 0.1 µg/mL amphotericin B + 0.6 µg/mL gentamicin B1.

### 2.4. Suppression of Antifungal Activity

The antibiotic activity against fungi is reduced by various environmental factors [13]. Similarly to the antibiotic effect of polyenes [19], such as the aminoglycoside tobramycin, which is lowered in the presence of Ca^2+^ ions [20], other factors could also reduce antifungal activity. Heparin reduced the antibiotic effect of neomycin [21].

The ionic antagonism weakened the antifungal effect of gentamicin B1, polymyxin, hygromycin and to a lesser extent clotrimazole and amphotericin B. Correspondingly, the MIC values of antifungal agents increased when fungal cells grew in the presence of these inhibitors. The MIC values of gentamicin B1 increased from 0.4 to 25 µg/mL in the presence of 1mg/mL CaCl_2_ against *Fusarium solani* and *Cryptococcus neoformans* cell cultures.

In Table 2 MIC values are presented in µg/mL representing the antifusarial and anticryptococcal activity of antifungal agents in the presence of inhibitors including heparin (1 mg/mL), CaCl_2_ (1 mM) and human serum (20%).

### 2.5. In Vivo Antifungal Effect

The results suggested that despite the inhibition by Ca^2+^, heparin or human serum, a significant antifungal activity remained after the in vitro reduction of gentamicin B1 that merited a test of its antifungal effect in vivo.

The time of survival of mice infected with *Cryptococcus* extended upon treatment with gentamicin B1 or amphotericin B relative to the infected but untreated control (Table 3). The intravenous (*i.v*.) administration of 5 mg/kg amphotericin B alone turned out to provide efficient protection, whereas the same i.v. dose of gentamicin B1 extended only moderately the survival of *Cryptococcus*-infected mice.

Higher concentrations of gentamicin B1 administered i.v. (≥25 mg/kg) were toxic when infected with *Cryptococcus neoformans* as judged by the survival of animals. The subcutaneous (*s.c*.) administration of gentamicin B1 at 10 mg/kg did not provide protection against cryptococcosis but an increased s.c., 50 mg/kg dose, moderately prolonged the survival of mice. The in vivo nephrotoxicity of amphotericin B is well known. Similarly, the in vivo toxicity of gentamicin B1 was found at higher concentrations.

Reduction of in vivo toxicity of amphotericin B and gentamicin B1 administering them together. Treatment is described in the Methods.

## 3. Discussion

The antifusarial effect of gentamicins and aminoglycoside derivatives was published recently [5]. The amphiphilic aminoglycosides particularly kanamycins attracted considerable interest for reviving these old drugs and by structural modifications turning them into antifungal agents [8]. They offer new options for fighting fungal pathogens and are examples of how to confront new therapeutic challenges [8,22,23,24]. The antibacterial to antifungal conversion of aminoglycosides goes back to the alkyl modification of old drugs, turning them into agrofungicides [25]. Most of the aminoglycosides, including gentamicin, consist of ring moieties with the amino-modified glycoside sugar (purpuroseamine) responsible for the binding to ribosomes and inhibiting protein synthesis [26]. The 2-deoxystreptamine-containing aminoglycosides (gentamicin, hygromycin, paromomycin, tobramycin, nebramycin, sisomicin) [5] are inhibitors of plant pathogenic fungi. Streptomycin lacking the 2-deoxystreptamine ring is not responsible for its antifungal effect, rather the substituents of its purpuroseamine ring could account for the antifungal activity.

Among the purpuroseamine substituents of aminoglycosides, the most efficient inhibitors of antifusarial activities point to the importance of the hydroxy and amino groups. The lowest MIC value was attributable to the 2,3,4-hydroxyl-5-aminoethyl- purpuroseamine substitutions of the hydroxy and aminoglycoside gentamicin B1. This value is between 0.4 and 3.1 µg/mL against *Fusarium solani,* two orders of magnitude below the murine in vivo toxicity of gentamicin.

To summarize the results, the gentamicin B1 fraction of the gentamicin complex possesses a new and robust antifungal effect broadening the spectrum of the already known antibacterial, antiprotozoal, anthelmintic gentamicins, reflecting a family of growth-controlling compounds. Gentamicin B1 exerts its selective fungistatic activity primarily on filamentous plant-pathogenic fungi, moderately inhibiting the growth of dermatophytes. As far as yeasts are concerned, *Candida albicans* is tolerant but *Cryptococcus* is sensitive to gentamicin B1.

The in vitro antifungal activity of gentamicin B1 is reduced in the presence of Ca^2+^, ions, heparin and human serum. Similarly to the antibiotic effect of polyenes [19] such as the aminoglycoside tobramycin, that is lowered in the presence of Ca^2+^ ions [21] other factors could also reduce the antifungal activity of aminoglycosides.

Regarding the structure–function relationship, the 2, 3, 4 and 5 substituents of the purpuroseamine ring of gentamicin B1 seem to be essential. The amino, methyl and hydroxy substituents are likely to impact the hydrophilic/lipophilic ratio and the antifungal character of gentamicins. The low in vitro MIC values show that gentamicin B1 and related structures merit further study as regards hemo- and cytotoxicity not only in inhibitory but also in the killing range. Gentamicin is in medical use and its minor fraction, gentamicin B1, could be tested against systemic plant mycoses that have recently had no efficient medical treatment. Another issue is the interaction between gentamicin B1 and amphotericin B on a chemical level, studying the exact mechanism of action.

Gentamicin B1 could be tested against *Fusarium* and *Aspergillus* infections even if considerable in vivo inactivation takes place. The longest survival of mice was achieved when the treatment of i.v. 5 mg/kg amphotericin B and i.v. 5 mg/kg gentamicin B1 was combined. Based on murine in vivo data, human doses of gentamicin B1 and the most favorable synergistic combinations with other compounds could reduce the toxic burden of other more toxic antifungal agents. As gentamicin is in medical use its minor fraction, gentamicin B1 could be immediately used against systemic plant mycoses that have recently had no efficient medical treatment. Combined gentamicin B1 and amphotericin B treatment as well as their repeated application at lower concentrations can reduce toxicity and improve the survival of patients.

## 4. Materials and Methods

### 4.1. Materials

Gentamicin C complex-producing *Micromonospora purpurea* var. *nigrescens* strain [27] was isolated in the Research Institute of Pharmaceutical Chemistry, (GYOKI), Budapest, Hungary. Gentamicin fractions were separated and purified to homogeneity by column chromatography. The purified gentamicin fractions were characterized, and their structures determined [28,29].

All industrial fungal and bacterial strains belonged to the GYOKI, Stock Culture Collection (GYOKI-SCC). Gentamicin fractions were obtained from Dr Janos Berdy, Department of Preparative Chemistry, Research Institute of Pharmaceutical Chemistry, Hungary. The antimicrobial activity of gentamicin fractions was tested at the Department of Chemotherapy and Antibiotics and murine experiments were carried out in the Animal Facility of the same Institute.

### 4.2. Sabouraud Broth

For more susceptible measurements, dilutions of antifungal agents were made with two-fold serial dilutions of sterile Sabouraud broth (Sigma-Aldrich, Budapest, Hungary) (pH 7.2) containing 3% α-d-glucose, 0.5% Witte’s peptone and 0.5% enzymatically digested casein in sterile distilled water. Antifungal agents were dissolved in a mildly heated mixture of 5 mL absolute alcohol and 5 mL sterile distilled water. The stock solution was diluted further with broth to less than 2.5% ethanol concentration, which has no inhibitory effect on cell growth. To determine MIC, serial dilutions ranging between 100 and 0.2 µg/mL were used. For microbial infection, 0.3 mL inoculum was added to 2.7 mL broth. Incubations lasted for six days at 34 °C.

### 4.3. Inocula

Sabouraud broth containing 0.5% Witte’s peptone, 2% glucose and 1.8% agar ((Sigma-Aldrich, Budapest, Hungary), in distilled water was autoclaved at 121 °C for 20 min at pH 6.0 and was used to prepare agar slant tubes. Streaking to plate out cells across the surface of the agar slants took place with the sterile loop of the wire inoculator containing a drop of cell culture. Fungal cells were incubated for six days at 34 °C. Spores were removed with 50 mL physiological salt solution (saline) and completed with saline to 100 mL, shaken for 24 h at 28 °C to get homogeneous distribution. After counting the living cell number (e.g., *Fusarium*), cell suspensions were stored in a refrigerator at 5 °C until further use. For infection, the initial germ number of the suspension for *Fusarium* and *Trichophyton* species was diluted to 10^5^ spores/mL, for *Microsporum gypseum* 10^6^ spores/mL. For the other fungi, including the yeast *Candida* species, the initial cell number was set to 2 × 10^4^ cells/mL. In vitro incubations in duplicate samples lasted for six days at 34 °C.

### 4.4. In Vitro Tests

Experiments were carried out with Sabouraud dextrose agar (Sigma-Aldrich, Budapest, Hungary) fungal medium at pH 7.2 by use of the two-fold dilution technique between 100 and 0.2 µg/mL concentrations of antifungal compounds. Minimal inhibitory concentration (MIC) was the lowest concentration where growth was not yet visible.

### 4.5. Animal Experiments

Female OF-1 mice (18–22 g) were from Charles River Laboratories. Mice were housed at room temperature and received laboratory mouse pellet diet and water ad libitum. Groups of mice consisted of ten female animals. Mice received humane care according to the criteria outlined by the Animal Research Guidelines of the Pharmaceutical Research Institute. Special permission for animal experiments was not needed in the year 1973, when these results were obtained.

In therapy studies, single i.v. or s.c. antifungal doses in 0.5 mL saline were administered at the same time as the cryptococcal challenge of mice. *Cryptococcus neoformans A-15053* infections were performed by the i.v. injection of 10^7^ yeast cells in 0.5 mL saline. More than 90% of untreated controls died in 6 days. Treatments were regarded as toxic when mice died at or before day 6. Survival of animals was registered up to two weeks.

## Figures and Tables

**Table 1:** *In vitro* activity of antifungal agents against plant pathogenic fungi.

Compound	Fungal Strain	MIC (µg/mL)
**Gentamicin B1**	*Fusarium solani*	0.4
**Gentamicin B1**	*Aspergillus flavus*	0.4
**Gentamicin B1**	*Aspergillus niger*	3.1
Clotrimazol	*Fusarium solani*	12.5
Amphotericin B	*Fusarium solani*	12.5
Nystatin	*Fusarium solani*	50
Griseofulvin	*Fusarium solani*	100
**Gentamicin B1**	*Microsporon gypseum*	3.1
Clotrimazol	*Microsporon gypseum*	6.2
Amphotericin B	*Microsporon gypseum*	50
Nystatin	*Microsporon gypseum*	50
Griseofulvin	*Microsporon gypseum*	100
**Gentamicin B1**	*C. neoformans A-15053*	*0.4*
**Clotrimazol**	*C. neoformans A-15053*	*0.4*
Amphotericin B	*C. neoformans A-15053*	*1.6*
Nystatin	*C. neoformans A-15053*	12.5
Griseofulvin	*C. neoformans A-15053*	50
**Clotrimazol**	*Trychophyton gypseum*	0.4
Amphotericin B	*Trychophyton gypseum*	6.2
Nystatin	*Trychophyton gypseum*	12.5
Gentamicin B1	*Trychophyton gypseum*	25
Griseofulvin	*Trychophyton gypseum*	50
**Amphotericin B**	*Candida albicans IAM 4888*	1.6
Clotrimazol	*Candida albicans IAM 4888*	*3.1*
Nystatin	*Candida albicans IAM 4888*	12.5
Gentamicin B1	*Candida albicans IAM 4888*	50
Griseofulvin	*Candida albicans IAM 4888*	100

**Table 2:** Suppression of *in vitro* antifungal activity of antifungal agents against *Fusarium* and *Cryptococcus* species.

Antifungal Agent	Suppression of Antifungal Activity (µg/mL)							
	*Fusarium solani*				*Cryptococcus neoformans*			
	Control	CaCl_2_	Heparin	Human Serum	Control	CaCl_2_	Heparin	Human Serum
		1 mg/mL	1 mg/mL	20% *v*/*v*		1 mg/mL	1 mg/mL	20% *v*/*v*
Gentamicin B1	0.4	25	50	3.1	0.4	25	50	0.8
Hygromycin B	0.8	25	12.5	1.6	6.2	50	6.2	6.2
Polymixin B	1.6	50	50	3.1	1.6	25	>50	6.2
Clotrimazol	12.5	25	50	25	0.4	0.4	0.4	0.8
Amphotericin B	12.5	>50	12.5	50	0.8	0.8	1.6	0.8

**Table 3:** *In vivo* anticryptococcal activity of amphotericin B and gentamicin B1 in mice.

No.	Antifungal Agent	Type of Treatment	Dose mg/kg	No. of Mice	No. of Animals Survived (Days)						
					1	2	3	6	8	10	14
1	Control	None	0	10	10	6	2	0			
2	Amphotericin B	*i*.*v*.	1	10	10	10	7	3	0		
3		*i*.*v*.	5	10	10	10	10	7	4	3	0
4	Amphotericin B	*s*.*c*.	2	10	10	9	6	0			
5		*s*.*c*.	10	10	10	10	10	5	5	4	2
6	Gentamicin B1	*i*.*v*.	5	10	10	6	5	1	0		
7		*i*.*v*.	25	10	10	2	1	0			
8	Gentamicin B1	*s.c.*	10	10	10	6	4	0			
9		*s.c.*	50	10	10	6	6	3	2	1	1
10	Gentamicin B1 + Amphotericin B	*i*.*v*.	5	10	10	10	10	10	10	5	4
		*i*.*v*.	5								
11	Gentamicin B1 + Amphotericin B	*s.c.*	10	10	10	10	4	3	0		
		*i*.*v*.	1								
12	Gentamicin B1 + Amphotericin B	*s.c.*	50	10	10	10	5	3	2	0	
		*i*.*v*.	1								
